# Training the Next Generation of Global Health Scientists: A School of Appropriate Technology for Global Health

**DOI:** 10.1371/journal.pntd.0000279

**Published:** 2008-08-27

**Authors:** Peter J. Hotez

**Affiliations:** Sabin Vaccine Institute and Department of Microbiology, Immunology, and Tropical Medicine, George Washington University Medical Center, Washington, D.C., United States of America

In March of 2008, the Bill & Melinda Gates Foundation made the impressive announcement that it will accept proposals for a new Grand Challenges Explorations program [1]. Grand Challenges Explorations will provide $100 million for global health scientists to identify new ways to protect against infectious diseases (including neglected tropical diseases [NTDs]), to create new drugs or delivery systems, to prevent or cure HIV/AIDS, and to explore the basis of latency in tuberculosis [1]. In so doing, the Gates Foundation will build on its long-standing multibillion dollar commitments to develop and test new drugs, diagnostics, and vaccines for NTDs, as well as the better known “big three” diseases, HIV/AIDS, tuberculosis, and malaria, and to fund critically needed operational research in support of large-scale control programs for these conditions [1]. The Gates Foundation is not alone—the United Kingdom's Wellcome Trust has a £15 billion investment portfolio of which a significant amount is devoted to global infectious diseases [2], while the United States National Institutes of Health (NIH) also devotes a significant amount of funding towards global health [3]. Therefore, in the coming decade we can expect that these initiatives will contribute significantly towards reducing the so-called *10/90 gap*, a term coined by the Global Forum for Health Research to refer to the finding that only 10% or less of the global expenditure on medical research and development is directed towards neglected health problems that disproportionately affect the poorest people in developing regions of sub-Saharan Africa, Asia, and tropical regions of the Americas.

While these new funded initiatives portend great promise for addressing the global 10/90 gap, I am also concerned that there is an important piece missing from these efforts that could undermine their success. I believe that at several levels we are failing to train the next generation of global public health scientists who either have the expertise to build these new technologies or who will understand how to apply these new technologies to public health practice [4]–[7]. The term *appropriate technology* has been used to describe innovations, which are “developed, produced, delivered and monitored within a comprehensive framework that takes into account the systems, the individuals, and the community.” [7]. In a previous paper, my co-authors and I cited as examples of appropriate technology the storied successes of vaccines for polio and measles, and how because of their appropriate use these diseases were eradicated from the Western Hemisphere; in the case of smallpox vaccine, we have achieved global eradication [7]. In contrast, the re-emergence of malaria in India and elsewhere as a consequence of chloroquine drug resistance by the malaria parasite and resistance to DDT by the mosquito vector are cited as examples of inappropriate use of technology during the 1960s [7],[8]. My concern is that unless we train a new generation of global health scientists to use appropriately the new Gates Foundation–Wellcome Trust–NIH–Howard Hughes Medical Institute–funded technologies, we risk seeing these impressive investments fail to materialize into innovations that actually make a difference in the lives of poor people in developing countries.

So exactly where and how does a person obtain training in a new type of global public health practice that embraces Affymetrix chips as a diagnostic tool for human African trypanosomiasis or river blindness; tests of a new drug for the treatment or chronic Chagas disease; planning for community-based drug distributors in order to add mebendazole and praziquantel to ivermectin for NTD control together with long-lasting insecticide-treated nets for co-endemic malaria; or the development, technology transfer, and clinical testing new recombinant vaccines for malaria, tuberculosis, or hookworm infection? Similarly, where and how does an individual become trained to simultaneously learn how to conduct high throughput screening for new antimalarial drugs, or prepare batch production records for the fermentation of a new recombinant hookworm antigen? In previous papers, I have suggested that if we had to answer the question today then we could not depend on most of the approximately 40 accredited public health schools in the United States [4]–[7]. Such statements were based partly on my personal experiences as a lecturer at two excellent public health schools (and my role as a visitor to several other schools) during which I was astonished to find that a majority of my students who were well into their public health training did not have a knowledge base that included the etiologic agent and mosquito vector of severe malaria, the differences between African and American trypanosomiasis, or even the differences between a drug or a vaccine, much less the technology that is required to build such control tools.

For me, it did not take long to discover why American students were so ill-equipped to take on substantive global health problems that require in-depth technical knowledge. In 2003, I reviewed the departmental structure of the first generation of US public health schools and compared it to some of the newer public health schools, which were established within the last 30 years [5]. Whereas the former under the influence of the great William Henry Welch included a heavy component of organism biology, pathology, and the state-of-the-art technologies of their day [4], many of the latter emphasize health promotion, health communication, health policy, and exercise sciences [5]. Although there is certainly nothing wrong with these important subjects, I feel it means that the pendulum of American public health education has swung heavily towards the social sciences, often at the expense of microbiology and infectious diseases [4]–[7]. In response, at The George Washington University and Sabin Vaccine Institute, I helped to create a new master's degree curriculum run jointly between our medical school's basic science Department of Microbiology, Immunology, and Tropical Medicine and the Department of Epidemiology and Biostatistics in our public health school. Even this initiative, however, falls short of what is truly needed to provide essential competencies in appropriate technology.

Since I first wrote those papers 5 years ago, which called for more technology-driven curricula, the situation has improved slightly. Competencies in the biologic basis of public health are now a core component of graduate training for the master's of public health degree [9], and as shown in Tables 1 and 2, there are currently 16 American public health schools with significant commitments to microbiology, infectious diseases, or biomedical sciences. Unfortunately, 11 of those schools represent the first generation of public health schools founded before 1970, while only five of the 25 American pubic health schools founded after 1970 host departments with capacity to begin addressing appropriate technology instruction. I feel that the two founding tropical medicine schools in the United Kingdom, the Liverpool School of Tropical Medicine and the London School of Hygiene and Tropical Medicine, come the closest to seriously addressing appropriate technology in their curricula [6], but even then they fall short in the areas of new tools development and testing. Certainly some of the new interdisciplinary global health training initiatives funded by the Fogarty International Center of the NIH, such as the Frameworks Programs for Global Health, will address some of these issues [10]. In the meantime, I am watching with great hope and expectation the founding of the new French School for Advanced Studies in Public Health [11].

**Table 1:** US Accredited Public Health Schools Founded before 1970 with Academic Departments Devoted to Infectious and Tropical Diseases.

Schools (Alphabetical Order)	Year Founded	Academic Department or Program
Harvard School of Public Health	1922	Immunology and Infection
Johns Hopkins Bloomberg School of Public Health	1916	Molecular Microbiology and Immunology
Tulane University School of Public Health and Tropical Medicine	1912	Tropical Medicine
University of California at Berkeley School of Public Health	1943	Infectious Diseases
University of Michigan School of Public Health	1941	Global and International Health
University of North Carolina at Chapel Hill School of Public Health	1936	Epidemiology
University of Pittsburgh Graduate School of Public Health	1948	Infectious Diseases and Microbiology
University of Texas School of Public Health	1969	Biomedical Lab Sciences
University of Washington School of Public Health and Community Medicine	1970	Pathobiology
Yale School of Public Health	1915	Epidemiology of Microbial Disease

Of the 15 accredited US public health schools, 11 host departments in this area.

**Table 2:** US Accredited Public Health Schools Founded after 1970 with Academic Departments Devoted to Infectious and Tropical Diseases.

Schools (Alphabetical Order)	Year Founded	Academic Department or Program
Emory University Rollins School of Public Health	1990	Hubert Department of Global Health
George Washington University Schools of Public Health and Health Service and Medicine and Health Sciences	1997	Public Health Microbiology and Emerging Infectious Diseases Program (jointly with Department of Microbiology, Immunology, and Tropical Medicine)
Instituto Nacional de Salud Publica (Cuernavaca, Mexico)	1987	Infectious Diseases Infectious Vector-Borne Diseases
University at Albany SUNY School of Public Health	1985	Department of Biomedical Sciences
University of South Florida	1984	Global Health Department

Of the 25 accredited US public health schools, five schools host departments devoted to this area.

At the turn of the 20th century William Henry Welch, who founded the original Johns Hopkins School of Public Health, teamed up with Wickliffe Rose, the architect of the Rockefeller International Health Board, in order to establish a plan for global public health education (the Rose–Welch Plan) that in some ways resembled the Flexner Report for medical education [4]. A new 21st century plan may be needed to address exactly how we might train a new generation of global public health scientists who will leverage the discoveries and technologies funded by Gates, Wellcome, Howard Hughes, and other foundations, as well as the NIH, the UK Medical Research Council, and their equivalent organizations in Brazil, China, India, and elsewhere.

Today, I feel that most of the innovation in global public health technology is coming not from schools of public health, but rather the public development partnerships (PDPs) and public–private partnerships (PPPs), many of which were launched with support from the Bill & Melinda Gates Foundation [12]–[14]. Listed in Table 3 are some of the major PDPs, which are producing an exciting new generation of products and developing control methods for the health of developing countries, and then introducing these innovations through a program of global access. As exciting as these new activities are, I am concerned that there is no mechanism to transfer the knowledge gained through them to the next generation of public health students.

**Table 3:** A Listing of the Major PDPs for Global Health.

Organization	Location
Program for Appropriate Technology in Health (PATH)	Seattle, WA, USA
PATH Malaria Vaccine Initiative	Bethesda, MD, USA
Medicines for Malaria Venture	Geneva, Switzerland
International AIDS Vaccine Initiative	New York, NY, USA
International Partnership for Microbiocides	Silver Spring, MD, USA
Aeras Global TB Vaccine Foundation	Rockville, MD, USA
Global Alliance for TB Drug Development	New York, NY, USA
Drugs for Neglected Diseases Initiative	Geneva, Switzerland, USA
Institute for One World Health	San Francisco, CA, USA
Foundation for Innovative New Diagnostics	Geneva, Switzerland, USA
Human Hookworm Vaccine Initiative- Sabin Vaccine Institute	Washington, D.C., USA
Infectious Disease Research Institute	Seattle, WA, USA
Pediatric Dengue Vaccine Initiative	Seoul, Korea
Innovative Vector Control Consortium	Liverpool, UK
UNC Consortium for Parasitic Drug Development	Chapel Hill, NC, USA
International Vaccine Institute	Seoul, Korea
PATH Vaccine Solutions	Washington, D.C. and Seattle , WA, USA
PATH Pneumococcal Vaccine Development Program	Washington, D.C. and Seattle , WA, USA
PATH Influenza Vaccine Development Program	Washington, D.C. and Seattle , WA, USA
PATH Advancing Rotavirus Vaccine Development	Washington, D.C. and Seattle , WA, USA
PATH Enteric Vaccine Initiative	Washington, D.C. and Seattle , WA, USA
PATH Meningitis Vaccine Project	Washington, D.C. and Seattle , WA, USA
WHO-TDR	Geneva, Switzerland

As the head of one such PDP, I have experienced great frustration with the lack of appropriate training of newly minted public health graduates. Among the core competencies that my PDP and others require are 1) the ability to recognize the major infectious pathogens with a microscope; cultivation and in vitro maintenance of organisms; and performance of diagnostic tests; 2) principles of drug discovery and high throughput screening; 3) principles of vaccine development including antigen discovery, fermentation/purification technology, and principles of quality control and quality assurances; 4) GXP (current good laboratory practices [GLP], good manufacturing practices [GMP], and good clinical practices [GCP]); 5) principles of promoting global access, such as technology transfer to innovative developing countries [15], product introduction, cost-effectiveness, and financing; 6) regulatory requirements for new drugs, diagnostics, and biologics; 7) international patent law; 8) basics of clinical trial design, mathematical principles essential to understanding transmission dynamics of disease, and translational epidemiology; and 9) the major operational research issues associated with large-scale infectious diseases control programs [7]. Most of these topics are covered in *PLoS Neglected Tropical Diseases*, although it should be pointed out that many of these core competencies would also be useful for addressing the ever-increasing burden of chronic non-communicable diseases in middle- and low-income countries [16].

The MPH degree, while important for many aspects of public health, is simply not adequate for providing most of the the skills we need to staff the PDPs and PPPs for global health technology innovation. We need additional programs of instruction in US schools of public health, and possibly a new type of *school of appropriate technology for global health*. Graduates of such a curriculum should be highly sought after by the PDPs and PPPs, as well as the pharmaceutical industry, NIH, US Centers for Disease Control and Prevention, and the World Health Organization and other United Nations agencies committed to global health. It may be that even the establishment of a single major school of appropriate technology for global health would be sufficient to begin addressing current needs, and then elements of such instruction would in time diffuse to more conventional schools of public health. Equally important, we need to address training needs in middle- and low-income countries where the neglected diseases are endemic by identifying centers of excellence in global health technology and expanding the opportunities for young scientists to obtain training in core competencies relevant to product and clinical development, GXP, and global access. In so doing, we could maximize the growing capacity for innovation in developing countries [17].

In my opinion, there is unprecedented interest by young people to solve global public health problems of importance to the developing world. We need now to harness that youthful energy and channel it appropriately.
